# Calculating real-world travel routes instead of straight-line distance in the community response to out-of-hospital cardiac arrest

**DOI:** 10.1016/j.resplu.2021.100176

**Published:** 2021-11-09

**Authors:** Christopher M. Smith, Ranjit Lall, Robert Spaight, Rachael T. Fothergill, Terry Brown, Gavin D. Perkins

**Affiliations:** a Warwick Clinical Trials Unit, University of Warwick, Coventry CV4 7AL, UK; b East Midlands Ambulance Service NHS Trust, Nottingham NG8 6PY, UK; c Clinical Audit and Research Unit, London Ambulance Service NHS Trust, London SE1 8SD, UK

**Keywords:** Out-of-hospital cardiac arrest, Public-access Automated External Defibrillators, Bystanders, Volunteer first-responders, Geographical Information Systems

## Abstract

**Background:**

Using straight-line distance to estimate the proximity of public-access Automated External Defibrillators (AEDs) or volunteer first-responders to potential out-of-hospital cardiac arrests (OHCAs) does not reflect real-world travel distance. The difference between estimates may be an important consideration for bystanders and first-responders responding to OHCAs and may potentially impact patient outcome.

**Objectives:**

To explore how calculating real-world travel routes instead of using straight-line distance estimates might impact the community response to OHCA.

**Methods:**

We mapped 4355 OHCA (01/04/2016-31/03/2017) and 2677 AEDs in London (UK), and 1263 OHCA (18/06/2017-17/06/2018) and 4704 AEDs in East Midlands (UK) using ArcGIS mapping software. We determined the distance from OHCAs to the nearest AED using straight-line estimates and real-world travel routes. We mapped locations of potential OHCAs (London: n = 9065, 20/09/2019-22/03/2020; East Midlands: n = 7637, 20/09/2019-17/03/2020) for which volunteer first-responders were alerted by the GoodSAM mobile-phone app, and calculated response distance using straight-line estimates and real-world travel routes. We created Receiver Operating Characteristic (ROC) curves and calculated the Area Under the Curve (AUC) to determine if travel distance predicted whether or not a responder accepted an alert.

**Results:**

Real-world travel routes to the nearest AED were (median) 219 m longer (623 m vs 406 m) than straight-line estimates in London, and 211 m longer (568 m vs 357 m) in East Midlands. The identity of the nearest AED changed on 26% occasions in both areas when calculating real-world travel routes. GoodSAM responders’ real-world travel routes were (median) 222 m longer (601 m vs 379 m) in London, and 291 m longer (814 m vs 523 m) in East Midlands. AUC statistics for both areas demonstrated that neither straight-line nor real-world travel distance predicted whether or not a responder accepted an alert.

**Conclusions:**

Calculating real-world travel routes increases the estimated travel distance and time for those responding to OHCAs. Calculating straight-line distance may overestimate the benefit of the community response to OHCA.

## Highlights

• In out-of-hospital cardiac arrest, straight-line distance estimates substantially underestimated actual travel distance for bystanders retrieving a nearby public-access AED and for volunteer first-responders travelling to the scene.
• Using real-world travel estimates changed the identity of the nearest public-access AED in more than a quarter of out-of-hospital cardiac arrests.

## Introduction

Early cardiopulmonary resuscitation (CPR) and defibrillation using an Automated External Defibrillator (AED) improves survival from out-of-hospital cardiac arrest (OHCA).1., 2. Both can both be performed by members of the public.

Bystanders at an OHCA may be able to retrieve a nearby AED. Estimates about the effective coverage area of a public-access AED – the maximum distance from an AED that an OHCA can occur for its retrieval to impact outcome – vary, with studies suggesting distances up to 100 m3., 4. and 500 m.^5^ Data from local Emergency Medical Services (EMS) in England demonstrated an ‘operational AED retrieval radius’ (distance from an OHCA within which they would ask bystanders to retrieve an AED) between 100–600 m.^6^

However, using radius or straight-line distance does not reflect actual travel routes and will overestimate AED coverage.^6^ In Hong Kong, calculating actual walking distance increased the average distance from an OHCA to the nearest AED from 231 m to 545 m, and reduced the proportion of AEDs within 100 m from 30% to 11%.^7^ In Italy, the geographical area that an AED covered was similar comparing a 200 m walking distance with a 100 m radius.^8^ Travel modality and speed will also affect an AED’s effective coverage area.

Many local EMS in the UK have integrated the GoodSAM first-responder app, which alerts nearby trained volunteers if an OHCA is diagnosed during a 999 (emergency) call.^9^ The alerting radius is determined by each local EMS. On receiving an alert, GoodSAM responders can ‘accept’ the alert and travel to the patient, potentially delivering prompt CPR and defibrillation. Real-world travel distance may be important for responders considering accepting an alert.

The aim of this study was to explore how real-world travel route calculations rather than straight-line distance estimates might impact the community response to OHCA. We have presented data from two datasets about the potential effect on i) bystander travel distances when retrieving a public-access AED and ii) the response of GoodSAM first-responders alerted to a nearby OHCA.

## Methods

### Bystander travel distances for AED retrieval

We obtained 12 months’ location data for OHCAs in London (01/04/2016–30/03/2017) and East Midlands (18/06/2017–17/06/2018) from the Out-of-Hospital Cardiac Arrest Outcomes (OHCAO) registry at the University of Warwick. Address and postcode were available for London, and postcode for East Midlands.

We converted OHCA locations to Eastings and Northings using a freely available Batch Geocoder (UK Grid Reference Finder Batch Convert Tool: https://gridreferencefinder.com/batchConvert/batchConvert.php).

London Ambulance Service (LAS) provided locations of public-access AEDs known to them on 13/12/2017. East Midlands Ambulance Service (EMAS) did so on 28/02/2019. Both provided AED location as Eastings and Northings.

We mapped OHCA and AED locations using ArcGIS (version 10.5.1, ESRI, California, USA) Geographical Information Systems (GIS) software, using ‘OS Open Carto’ (Ordnance Survey Limited, Southampton, UK) as a ‘basemap’ (background map). This map provides coverage for Great Britain and uses the British National Grid coordinate system, onto which coordinates provided as Eastings and Northings can be displayed without needing spatial transformations.

We used ArcGIS’ Near Tool to identify the nearest AED to each OHCA and to calculate straight-line distance between the two. To calculate real-world travel distance, we inputted information from ‘OS Open Local’ Vector maps (Ordnance Survey Limited, Southampton, UK) for the relevant geographical areas. This map has roads as ‘vector’ features – lines that can be overlaid on the basemap – and we used ArcGIS to create a network from them. This allowed us to model travel routes along the network. We then used the Closest Facilities function of the Network Analyst Tool to determine the road/path travel distance (in metres) from each OHCA to its nearest AED. We calculated real-world travel-time using a speed of 100 m/min, following assessments by Deakin et al.^6^

### GoodSAM volunteer first-responders

We obtained six months’ data from GoodSAM alerts after diagnosis of potential OHCA in London (20/09/2019–22/03/2020) and East Midlands (20/09/2019–17/03/2020). We determined the proportion of incidents when GoodSAM responders accepted an alert; reached the scene; and reached the patient’s side before EMS.

For this 2019–20 dataset, GoodSAM provided both incident and responder location at the time of the alert as latitude and longitude. We converted these to Eastings and Northings using the UK Grid Reference Finder Batch Convert Tool and plotted them in ArcGIS on the OS Open Carto basemap as described above.

We used ArcGIS’ XY To Line Tool to match each GoodSAM responder location to its relevant incident and provide a straight-line distance between the matched points. To calculate real-world travel distance we again overlaid the road network from the OS Open Local Vector map. Using the Network Analyst Tool we matched GoodSAM responder location to its relevant incident using a common numerical identifier, and ArcGIS then provided a travel route between them. We calculated real-world travel-time estimates using a speed of 100 m/min.^6^

We created Receiver Operating Characteristic (ROC) curves and have presented Area Under the Curve (AUC) statistics to determine if there was an optimum distance threshold for alert acceptance and for reaching the patient before EMS in London and East Midlands, for both straight-line and real-world travel distance. For a responder’s distance from an incident, the ROC determines the likelihood of a ‘true positive’ (presented as the sensitivity: here, either alert acceptance or arrival at the scene before the ambulance) or a false positive (1-specificity: rejecting an alert or failing to reach scene). AUC values range from 0 (distance incorrectly predicts responder action in all cases) to 1 (distance correctly predicts responder action in all cases); a value of 0.5 suggests that distance predicts responder action no better than chance would.

In London, GoodSAM increased its alerting radius from 300 m to 700 m in July 2018. Knowing responder location for the 2019–20 London dataset meant that we could compare the current 700 m response radius with what the response *would have been* if the radius was still only 300 m.

We have presented travel distances and times as median with interquartile range (IQR) and used the related-sample Wilcoxan Signed Ranks test to compare the median of differences between straight-line and real-world travel distances. We used Pearson R to correlate differences between straight-line and real-world travel distances for bystander AED retrieval.

## Results

### Bystander travel distances for AED retrieval

**Fig. 1:**
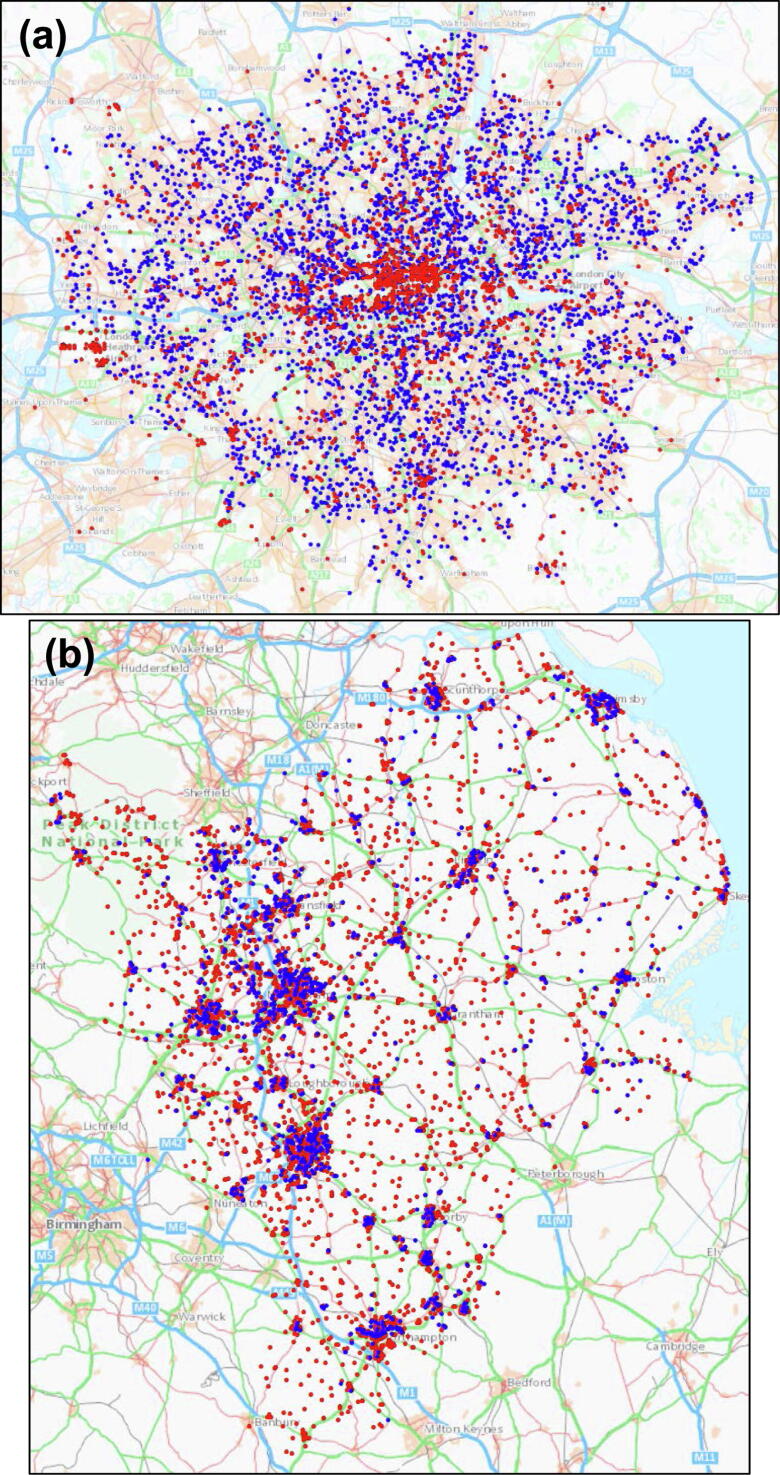
Locations of OHCAs (blue dots) and AEDs (red dots). (a) London; scale 1:250,000. (b) East Midlands; scale 1:800,000.

We had location data for 4355/4448 (98%) OHCAs in London and 1263/2281 (55%) OHCAs in East Midlands in the respective study periods. We mapped 2677 AEDs in London and 4704 AEDs in East Midlands. **(**Fig. 1**).**

In London OHCAs were a median of 406 m (IQR 223–643 m) from the nearest public-access AED by straight-line distance and 623 m (IQR 348–953 m) by real-world travel distance (difference in distance route; p < 0.0001). The median ratio of real-world to straight line distance was 1.39 (IQR 1.21–1.71). For a bystander at an OHCA this would be an extra travel distance of (median) 434 m (217 m there and back), equating to an extra (median) 04:20 minutes at a brisk walking speed of 100 m/min. Using real-world travel estimates, the number of OHCAs within 100 m of an AED reduced from 8.6% to 6.3%, and within 500 m from 61% to 39%.

In East Midlands OHCAs were a median of 357 m (IQR 201–557 m) from the nearest public-access AED by straight-line distance and 568 m (IQR 317–894 m) by real-world travel distance (difference in distance route; p < 0.0001) The median ratio of real-world to straight line distance was 1.42 (IQR 1.23–1.80). For a bystander at an OHCA this would be an extra travel distance of (median) 422 m, equating to an extra (median) 04:13 minutes. Using real-world travel estimates, the number of OHCAs within 100 m of an AED reduced from 8.3% to 5.5%, and within 500 m from 69% to 43%.

Table 1 further details the proximity of OHCAs to the nearest AED.

**Table 1:** Proximity of OHCAs to public-access AEDs.

	London Ambulance Service (n = 4355)				East Midlands Ambulance Service (n = 1263)			
	Straight-line distance		Real-world travel distance		Straight-line distance		Real-world travel distance	
<100 m	8.6%	(373)	6.3%	(273)	8.3%	(105)	5.5%	(70)
<200 m	22%	(951)	12%	(538)	25%	(315)	13%	(165)
<300 m	36%	(1568)	20%	(879)	41%	(514)	23%	(289)
<400 m	49%	(2136)	30%	(1288)	57%	(714)	34%	(428)
<500 m	61%	(2650)	39%	(1683)	69%	(867)	43%	(547)
<1000 m*	92%	(3989)	77%	(3358)	93%	(1171)	81%	(1024)

* The remaining OHCAs were > 1000 m from the nearest public-access AED.

**Fig. 2:**
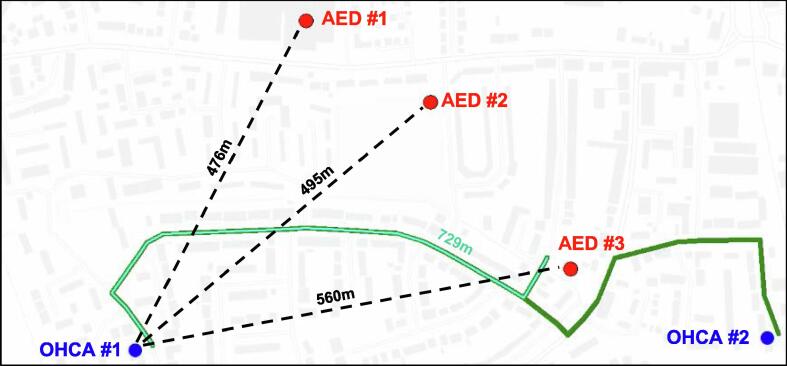
Real-world vs straight-line travel distances for two example OHCAs (blue dots) and three nearby AEDs (red dots). OHCA #1 is closest by straight-line distance (dashed line, 476 m) to AED #1, but closest by real-world travel distance (light-green solid line, 729 m) to AED #3.

When considering real-world rather than straight-line travel distance, the identity of the nearest public-access AED changed in 26% (1133/4355) cases in London, and in 26% (329/1263) cases in East Midlands. Fig. 2 illustrates an example of this.

**Fig. 3:**
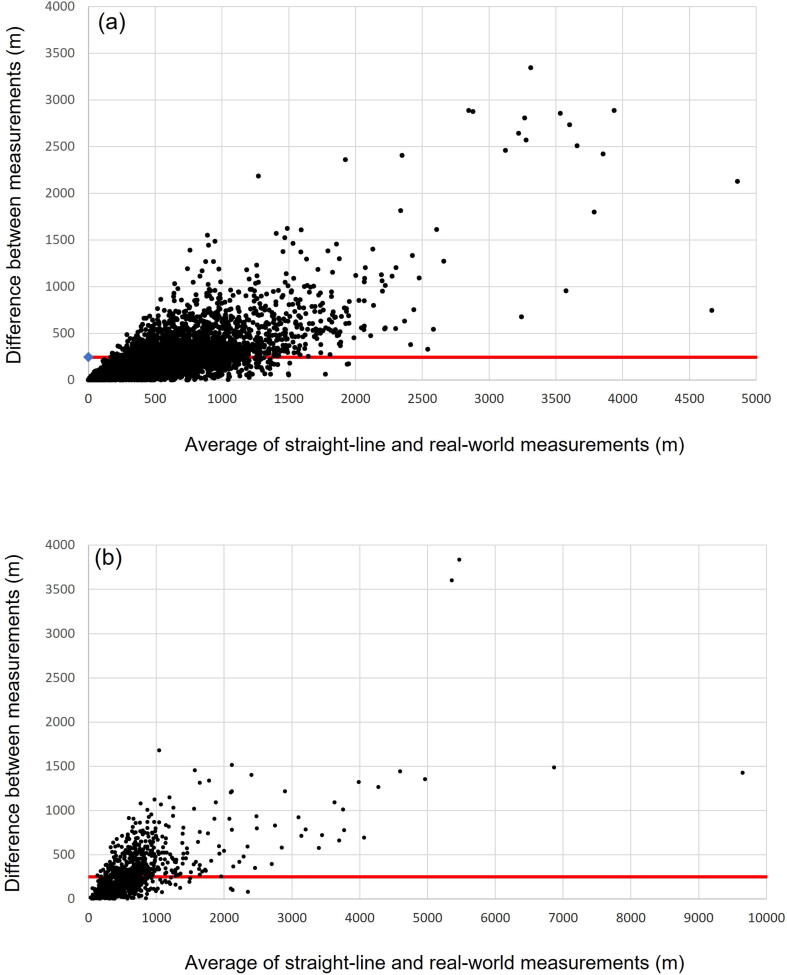
Bland-Altman plot, London (a) and East Midlands (b). Average difference between straight-line and real-world measurements is indicated by solid red line.

The average difference between straight-line and real-world distances was 243 m (standard deviation 270 m) in London and 247 m (standard deviation 282 m) in East Midlands. Fig. 3 (Bland-Altman plots) depict this further, showing closer agreement and less bias at smaller distances. The IQR for differences between straight-line and real-world distances was 76–322 m in London and 75–329 m in East Midlands. Overall, correlation between straight-line and real-world travel distance was high – Pearson R 0.933 (London) and 0.883 (East Midlands), both p < 0.001.

### GoodSAM volunteer first-responders

**Fig. 4:**
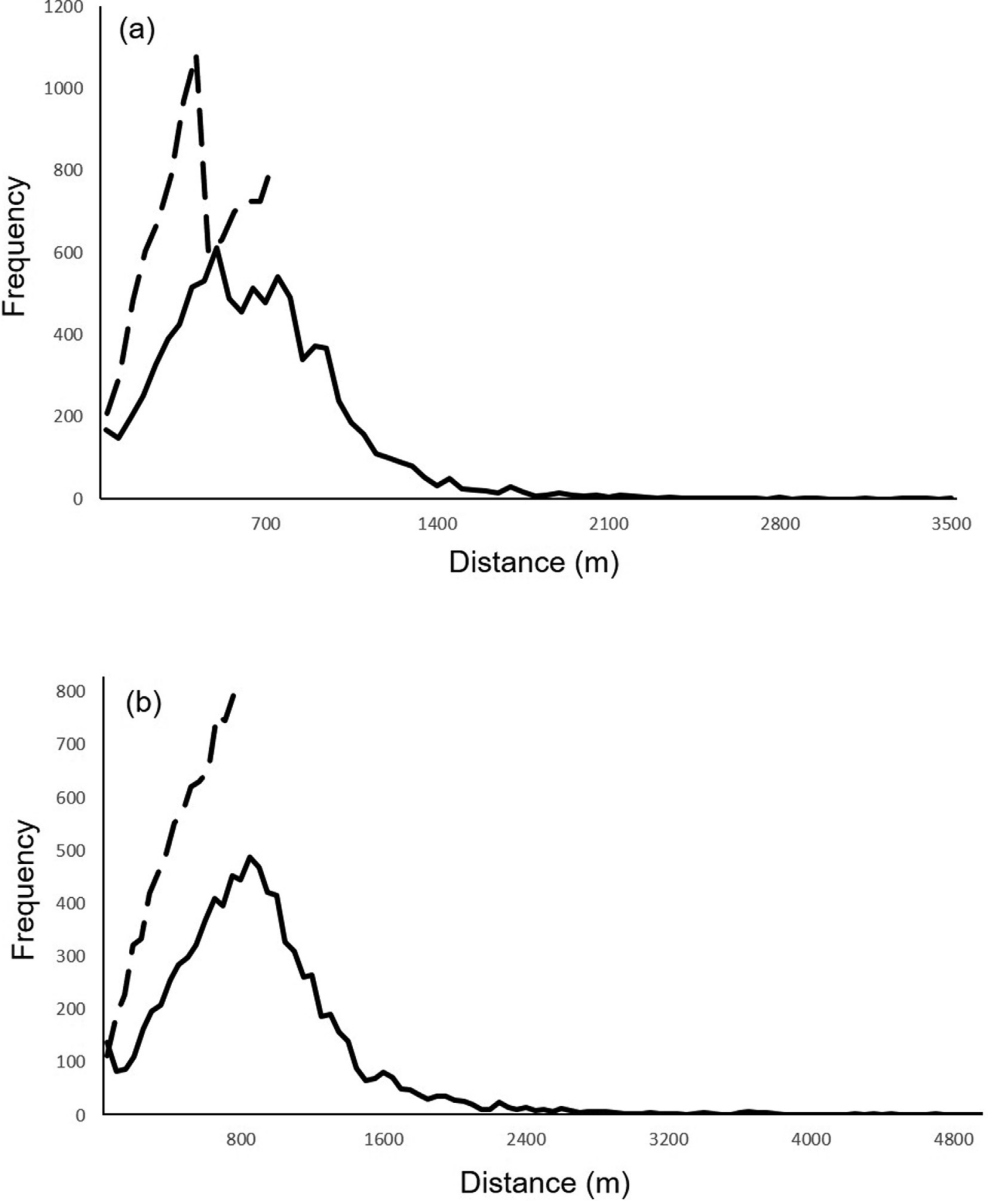
Frequency of alerting distance (dotted line) and real-world travel distance (solid line) for GoodSAM responders in (a) London and (b) East Midlands.

Fig. 4 depicts the distribution of alerting distance and GoodSAM responder travel distances following an alert.

London: There were 9180 alerts for 4776 incidents, and we calculated distance between GoodSAM responder and incident in 99% (9065/9180) cases.

GoodSAM responders accepted 23% (2088/9180) alerts. We had data for those reaching the scene in 1888 of 2088 cases: 95% (1800/1888) reached the scene. We had data for those reaching the patient’s side in 1781 cases: 41% (734/1781) arrived before EMS.

For the 9065 cases where we knew the distance between responder and incident, the median alerting radius (straight-line distance) was 379 m (IQR 255–548 m) and the median real-world travel distance was 601 m (IQR 388–826 m) (difference in distance route; p < 0.0001) – a ratio of real-world to straight line distance of 1.59. We estimated a median response time based on real-world travel routes of 6:01 min (IQR 3:53–8:16 min) at 100 m/min.

**Fig. 5:**
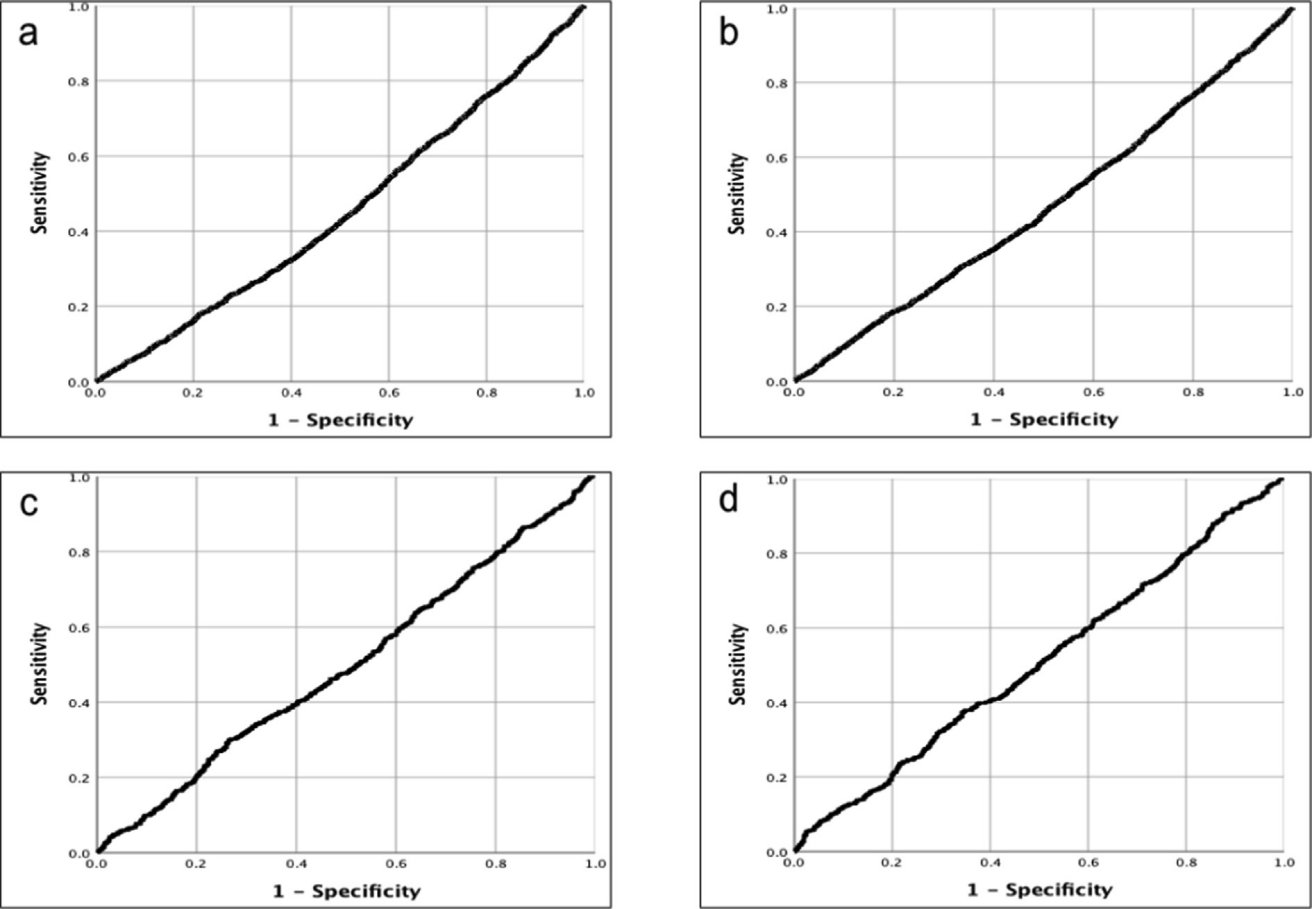
ROC curves, London. Relationship between a) alerting radius and alert acceptance; b) real-world travel distance and alert acceptance; c) alerting radius and arrival at patient before EMS; d) real-world travel distance and arrival at patient before EMS.

Neither alerting radius nor real-world travel distance predicted whether or not an alert was accepted (data from 9065/9180 cases where we knew the distance between responder and incident, AUC 0.454 and 0.469 respectively) or whether or not the responder reached the patient before EMS (data from 1638/1781 cases for those arriving before EMS where we knew the distance between responder and incident, AUC 0.497 and 0.506). Fig. 5 **s**hows the ROC curves.

Using the current 700 m alerting radius meant far more alerts were accepted than would have been using the previous 300 m radius (2038 vs 744) with a small decrease in alert acceptance *rate* to 22% (2038/9065) from 25% (744/2967) (odds ratio (OR) 0.87, 95% CI 0.79–0.95; p = 0.004). The proportion of people reaching the scene and reaching the patient before EMS was similar for both the 700 m and 300 m alerting radius, where this was known (reaching scene: 95% (1754/1841) vs 95% (644/678), OR 1.06, 95% CI 0.71–1.60, p = 0.76; reaching patient before EMS 39% (718/1841) vs 38% (255/678), OR 1.06, 95% CI 0.88–1.27, p = 0.53). However, the *absolute* numbers of responders reaching the scene (1754 vs 644) and reaching the patient before EMS (718 vs 255) were substantially higher with the 700 m alerting radius.

East Midlands: There were 7741 alerts for 4177 incidents, and we calculated distance between GoodSAM responder and incident in 99% (7637/7741) cases.

GoodSAM responders accepted 29% (2252/7741) alerts. We had data for those reaching the scene in 1946 of 2252 cases: 94% (1824/1946) reached the scene. We had data for those reaching the patient’s side in 1803 cases: 68% (1227/1803) arrived before EMS.

For the 7637 cases where we knew the distance between responder and incident, the median alerting radius was 523 m (IQR 341–773 m) and the median real-world travel distance was 814 m (IQR 553–1077 m) (difference in distance route; p < 0.0001) – a ratio of real-world to straight line distance of 1.56. We estimated a median response time based on real-world travel routes of 8:08 min (IQR 5:32–10:46 min) at 100 m/min.

**Fig. 6:**
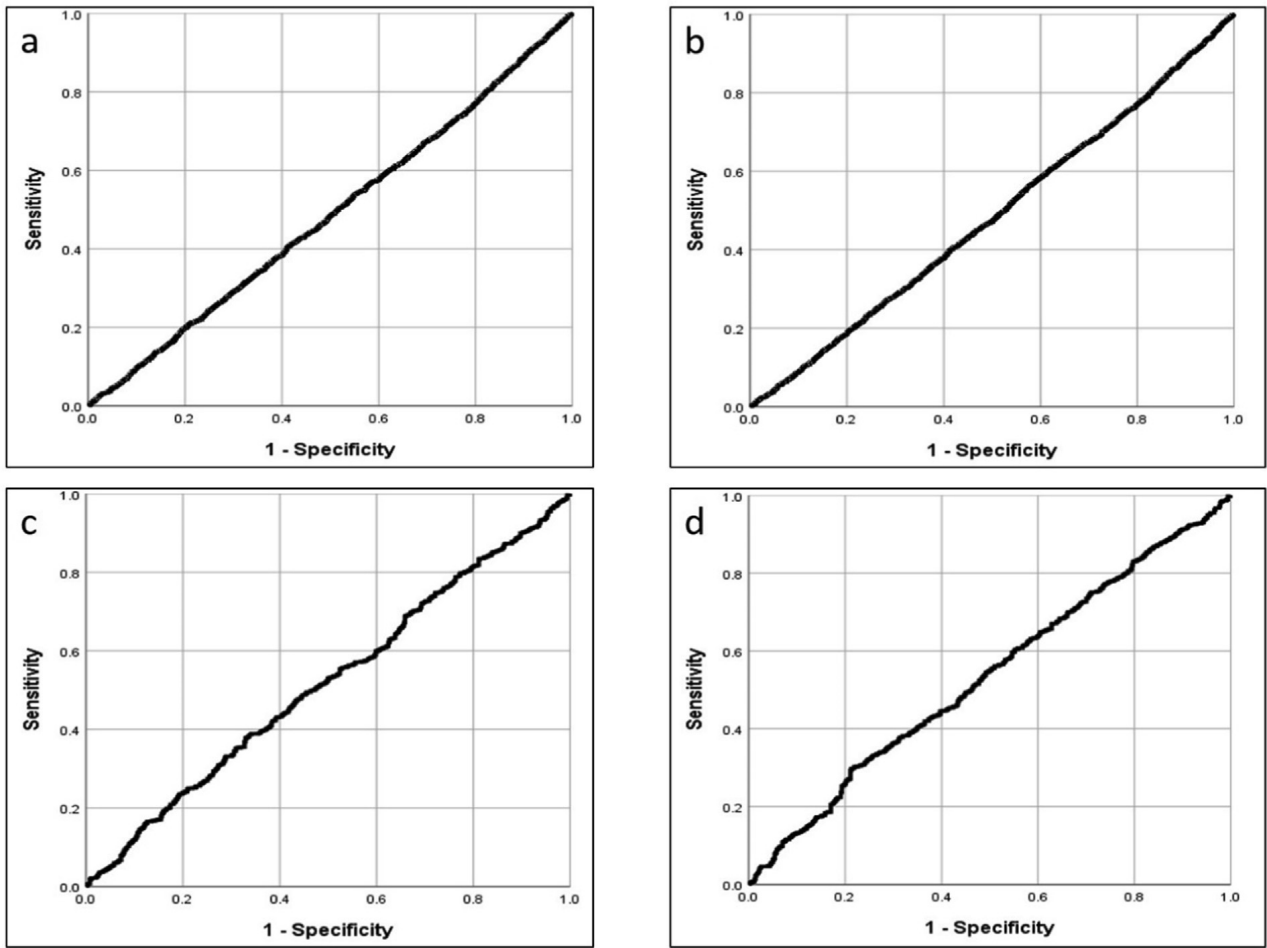
ROC curves, East Midlands. Relationship between a) alerting radius and alert acceptance; b) real-world travel distance and alert acceptance; c) alerting radius and arrival at patient before EMS; d) real-world travel distance and arrival at patient before EMS.

Neither alerting radius nor real-world travel distance predicted whether or not an alert was accepted (data from 7637/7741 cases where we knew the distance between responder and incident, AUC 0.486 and 0.483 respectively) or whether or not the responder reached the patient before EMS (data from 1720/1803 cases for those arriving before EMS where we knew the distance between responder and incident, AUC 0.517 and 0.533). Fig. 6 shows the ROC curves.

## Discussion

### Main findings

In this study, bystanders at an OHCA would have to travel more than 400 m further – or for more than four minutes longer (median values) – to retrieve a public-access AED in London and East Midlands than estimated by straight-line distance. In both regions, using real-world travel routes changed the identity of the nearest AED in 26% of cases.

In addition to real-world distances being, on average, significantly longer than straight-line distances, an important finding from our paper is the high level of variation between individual OHCA cases in the discrepancy between the two distance measures. This has important implications for the precision of response-time estimates, in that the use of straight-line distances would increase the level of statistical noise relative to real-world distances. Ideally, effective AED coverage areas and response distances should not be based on simple radius and could be tailored for different locations (e.g. urban vs suburban) within EMS regions.

GoodSAM responders’ real-world travel distances were a median of 222 m (London) and 291 m (East Midlands) longer than the straight-line distance estimates. Importantly, neither alerting distance (a straight-line/radius predetermined by *local EMS*) nor responder travel distance (a real-world travel distance estimate based on responders’ travel routes) predicted alert acceptance better than chance.

In London, increasing the alerting radius from 300 m to 700 m had little effect on the *proportion* of responders accepting an alert, reaching the scene, or reaching the patient before EMS. However, the *absolute* numbers substantially increased, thus increasing the opportunity for responders to provide meaningful intervention before the arrival of EMS.

### Comparison with the literature

The proportion of OHCAs within 100 m (6.3% London and 5.5% East Midlands) and 500 m (39% London and 43% East Midlands) of the nearest public-access AED are similar to those reported in the South Central Ambulance Service region (2014–2016: 5.9% <100 m and 36% <500 m).^6^ Researchers in Hong Kong demonstrated substantially increased travel distances between 5119 historical OHCAs and 1637 public-access AEDs using real-world estimates rather than straight-line estimates. The average increased from 231 m to 543 m.^7^

This is the first study to our knowledge to report that the identity of the nearest public-access AED to an OHCA often changes when calculating real-world travel routes. We also believe that this is the first study that reports and quantifies the difference between a volunteer first-responder’s straight-line distance and their actual real-world travel distance, and the first time that there has been any comparison between two different alerting radii (300 m vs 700 m here) in the same system.

### Strengths and limitations

We were only able to accurately plot locations for 55% OHCAs in East Midlands, as EMAS routinely provided only a Utstein definition^10^ for location for part of the study period. Even in these cases there will have been some imprecision in the location as we only had postcode: this is shared by an average of 15 properties, although this can vary markedly dependent on area and housing density.^11^ Findings were similar to those in London, and we think it important to present the results of these analyses here having performed the study. However, we fully acknowledge the uncertainty caused by the large amount of missing data, and that results from this dataset should be interpreted with caution.

The findings relating to bystander AED retrieval represent the potential for public-access AED use in ideal situations. AEDs may not have been available or accessible for use at the time of the OHCA, but we were unable to capture this data. Previous results from the UK^6^ and internationally^12^ suggest out-of-hours availability is substantially reduced. There may often be public-access AEDs unknown to EMS, which might be brought by knowledgeable bystanders to the scene.^13^

Bystanders and GoodSAM responders may feasibly travel at faster than walking pace, or by other travel modalities, during an emergency response. Although the maps used are detailed and show all roads as well as other marked walking and cycling paths, we anticipate that there may be short-cuts or other unmapped routes that could shorten travel distance. Thus, travel-time estimates may be pessimistic. However, we also did not factor in time taken to find an AED or the incident once in the right location.

It is unintuitive that an increasing travel distance for GoodSAM first-responders is not predictive of alert acceptance. We did not examine how incident characteristics might affect response decisions – when alerted the responder will not have detailed information about the incident or patient. We did not have information about responders themselves or other factors at the time of the alert, and there are a number of other behavioural factors that may affect the likelihood that a responder will accept an alert and travel to the patient.^15^ This, and other unexplored confounding factors might help explain why we did not demonstrate a simple relationship between travel distance and likelihood of accepting an alert.

## Clinical implications and recommendations

The increase in travel-time estimates by calculating real-world travel routes, either to retrieve an AED or when acting as volunteer first-responder, may be clinically relevant and affect patient outcomes. Researchers have modelled optimal placement of public-access AEDs based on OHCA location and incidence,4., 14. but future modelling should ideally account for increased retrieval distance and time estimates using real-world travel routes. If local EMS use operational AED retrieval distances based on straight-line estimates, they will overestimate how often an AED can successfully be retrieved. They will not always direct the bystander to the nearest device.

Our findings suggest it is worthwhile exploring a strategy of alerting more responders by increasing alerting distance. However, there may be a threshold beyond which the number of alerts a responder receives over time is so high, and the likelihood of them reaching the patient before EMS is so low, that they are de-motivated from responding to the current and future alerts. We did not find that limit here, but it remains an important consideration for this and for similar volunteer first-responder systems worldwide.

## Ethics Information

The University of Warwick Biomedical and Scientific Research Ethics Committee granted ethical approval on 06/03/2018 (London data) and 29/10/2018 (East Midlands) for work related to bystander public-access AED retrieval (REGO-2018-2157), and 05/06/2019 for work related to GoodSAM first-responders (BSREC-50/18-19).

The OHCAO registry has approval from the Health Research Authority (ref:13/SC/036) and Confidentiality Advisory Group (ref:ECC8-04(C)/2013). The registry granted approval for use of non-identifiable data for our analyses.

## Conclusion

Using real-world rather than straight-line distance estimates significantly increased bystander AED retrieval distance and time and changed the identity of the nearest public-access AED in a quarter of OHCAs. This has important implications for EMS directing bystanders at the scene to the nearest available AED.

Response distance did not predict whether or not a GoodSAM responder would accept an alert or reach the patient before EMS. Increasing the alerting radius in London from 300 m to 700 m substantially increased the number of patients that might benefit from a GoodSAM response, with little effect on the likelihood that responders would accept an alert or reach the patient before EMS. We did not find an upper effective limit for the alerting radius.

## CRediT authorship contribution statement

**Christopher M. Smith:** Conceptualization, Formal analysis, Funding acquisition, Investigation, Methodology, Project administration, Writing – original draft, Writing – review & editing, Visualization. **Ranjit Lall:** Conceptualization, Formal analysis, Methodology, Writing – review & editing. **Robert Spaight:** Conceptualization, Resources, Writing – review & editing. **Rachael T. Fothergill:** Conceptualization, Resources, Writing – review & editing. **Terry Brown:** Methodology, Writing – review & editing. **Gavin D. Perkins:** Conceptualization, Funding acquisition, Writing – review & editing, Supervision.
